# Regulatory‐Driven Pharmacokinetic and Immunogenicity Study of Siltartoxatug, a Recombinant Native Human Monoclonal Antibody Against Tetanus Toxin, in Cynomolgus Monkeys

**DOI:** 10.1155/adpp/2804371

**Published:** 2026-10-18

**Authors:** Xia Zhou, Fei Zhou, Zhenpeng Fu, Weijun Ye, Guo Li, Jason Li, Weihong Zheng, Hua-Xin Liao, Shaojie Wang, Xiaoyi Liu

**Affiliations:** ^1^ School of Pharmaceutical Engineering Shenyang Pharmaceutical University Shenyang 110016 Liaoning China syphu.edu.cn; ^2^ Zhuhai Trinomab Pharmaceutical Co., Ltd. Zhuhai Guangdong China

**Keywords:** anti-drug antibody (ADA), bioanalysis, immunogenicity, monoclonal antibody, pharmacokinetics, siltartoxatug, tetanus

## Abstract

Siltartoxatug—developed by Trinomab Pharmaceutical Co., Ltd., using its proprietary HitmAb® platform—is the first‐in‐class recombinant monoclonal antibody drug for urgent prophylaxis of tetanus approved by China National Medical Products Administration in February 2025, which addresses the critical unmet need to substitute blood‐derived passive immunization preparations for prevention of tetanus. The nonclinical pharmacokinetic and immunogenicity study of siltartoxatug was designed and conducted in strict compliance with global regulatory guidelines for initiating the first‐in‐human clinical trials. Herein, we report the comprehensive findings from this study. Twenty‐four naive cynomolgus monkeys (*Macaca fascicularis*) were randomly assigned to four groups each with 6 animals. Monkeys in Group 1 received intravenous infusion of siltartoxatug at 1 mg/kg. Monkeys in Groups 2, 3, and 4 received intramuscular injection of siltartoxatug at dose of either 1 mg/kg, 3 mg/kg, or 10 mg/kg. Blood samples were collected over 57 days post‐administration, and serum drug concentrations and anti‐drug antibody levels were determined with validated methods. Results showed that intramuscular administration of siltartoxatug exhibited favorable pharmacokinetic characteristics: primarily systemic distribution with high bioavailability (88.0% at 1 mg/kg) and low apparent volume of distribution (58.7–69.4 mL/kg), prolonged elimination half‐life (11.7–12.4 days), short mean time to maximum concentration (1.2–2.7 days), and dose‐dependent systemic exposure without significant sex differences. Additionally, minimal anti‐drug antibody responses were observed. These findings underpinned greater predictability for its clinical translation and informed its first‐in‐human development.

## 1. Introduction

Tetanus is a serious and potentially fatal disease caused by infection with *Clostridium tetani*, a Gram‐positive obligate anaerobe. The bacterial spores enter the body through wounds and then germinate under anaerobic conditions to produce the tetanus neurotoxin (TeNT), a 150‐kDa protein consisting of three functional fragments (A, B, and C) that mediate neuron‐binding (fragment C), translocation (fragment B), and metalloproteinase activity (fragment A), respectively [1]. The toxin can disseminate systemically via lymphatic and blood circulation [2, 3]. Ultimately, the metalloproteinase activity of fragment A inhibits the neurotransmitter release, leading to muscle rigidity and spasms—the typical clinical manifestations of tetanus [4]. Neutralizing antibodies blocking the functions of TeNT are key effectors of tetanus prophylaxis. Tetanus toxoid (TT) vaccine, containing formalin‐inactivated TeNT that elicits robust endogenous antibody responses, is widely used for active immunization to achieve long‐term protection [5]. In contrast, passive immunization with preformed antitoxin antibodies is essential to provide rapid and assured protection for patients with incomplete or undocumented vaccination records [6]. Human blood–derived tetanus immune globulin (HTIG) is currently the most widely used passive immunization agent for tetanus worldwide, while the equine heterologous immunoglobulin still exists in clinical practice in India and China. Due to the inherent drawbacks, such as supply limitation and safety risks, of equine and human serum–derived tetanus immune globulin, highly potent recombinant neutralizing monoclonal antibodies (mAbs) against tetanus toxin may serve as a superior alternative [7].

Siltartoxatug, developed by Trinomab Pharmaceutical Co., Ltd., is derived from a native human mAb isolated from a single plasma B cell by using the proprietary human mAb technology HitmAb® platform. The mAb is produced as human IgG1 in Chinese hamster ovary (CHO) cells in large scale under Good Manufacturing Practice (GMP) condition. The molecule has a deglycosylated intact molecular weight of 144.2 kDa and an isoelectric point at 9.2. It has been postulated that the mechanism of action of siltartoxatug is to bind specifically to a conformational epitope consisting of critical amino acid residues on the fragments A and B of TeNT, resulting in blocking the translocation and metalloproteinase activity of TeNT [8]. In vivo toxin challenge tests in mice showed that siltartoxatug has a potency of 400 IU/mg [8], corresponding to ∼0.5–1.7 IU/mg for HTIG products [9, 10]. Such an exceptionally high neutralizing potency, combined with the lack of mAb‐based therapies for tetanus worldwide, makes it an attractive potential first‐in‐class innovative candidate for development as therapeutic drug against tetanus.

For first‐in‐class innovative drugs, regulatory agencies including European Medicines Agency (EMA), U.S. Food and Drug Administration (FDA), and China National Medical Products Administration (NMPA) require comprehensive animal pharmacokinetic (PK) and immunogenicity studies to support clinical development [11–13]. Core PK parameters (bioavailability, half‐life, clearance, and volume of distribution) are essential to predict the drug’s behavior in humans and formulate clinical trial strategies. Meanwhile, the immunogenicity study reveals how anti‐drug antibodies (ADAs) impact PK, efficacy, and safety in animal models. While these results cannot predict human immunogenicity, they help optimize ADA monitoring strategies for clinical trials.

Given the bridging and indicative value of animal studies for clinical research, ICH and regulatory authorities recommend [13–15] that the selection of animal models must be justified based on pharmacological similarity to humans. Since siltartoxatug specifically targets an exogenous molecule—tetanus toxin—rather than any known endogenous proteins in either humans or animals, no target‐related pharmacologically relevant animal species exist for this drug. However, cynomolgus monkeys are evolutionarily more closely related to humans than other animal species commonly employed in nonclinical studies [16]. Particularly, the binding affinities of human mAbs for cynomolgus monkey vs. human neonatal Fc receptor (FcRn) are comparable, and the relative rank order of binding and pH‐dependent interaction patterns are highly preserved between human and cynomolgus FcRn [17]. Therefore, cynomolgus monkeys were identified as the most suitable species for the nonclinical PK study of siltartoxatug.

Circulating antitoxin at protective levels is required to prevent tetanus in humans [18]. For urgent tetanus post‐exposure prophylaxis (PEP), rapid attainment of protective antibody levels—rather than immediate maximal peak concentration—is therefore critical. The intramuscular (IM) route offers faster systemic exposure than subcutaneous injection while avoiding the adverse events and resource burden of intravenous (IV) infusions, an advantage particularly valuable in emergency‐care settings [19]. A single IM dose of HTIG has been used successfully worldwide for decades and recommended by the World Health Organization (WHO) as the preferred passive tetanus immunotherapy [5]. While siltartoxatug is a recombinant monoclonal neutralizing antibody, it confers passive tetanus immunization, as does HTIG. Accordingly, a single IM injection was selected as the intended clinical regimen for siltartoxatug, consistent with established real‐world clinical practice. Therefore, single‐dose IM groups were adopted in this study. To minimize animal numbers following the 3R principles, only one IV group was included for the calculation of absolute bioavailability. Dose levels were justified based on multiple factors, primarily from murine efficacy data [8] and the established clinical dose of HTIG [10].

Herein, we present the PK study of siltartoxatug in cynomolgus monkeys, which was designed and conducted in strict compliance with global regulatory guidelines, with two specific objectives: (I) to characterize the serum PK profile of siltartoxatug in naive male and female cynomolgus monkeys following a single IM administration, by quantifying serum drug concentrations up to 57 days post‐dosing, and (II) to assess its immunogenicity via measuring serum ADA.

## 2. Materials and Methods

### 2.1. Test Materials

Siltartoxatug (Batch Nos. 2230W191212Y and 2230WD191226K01X01D01) was manufactured by WuXi Biologics. The drug product is formulated in a histidine/histidine hydrochloride buffer system containing sucrose, sodium chloride, and polysorbate 80, with a pH value of 6.0.

Dosing solutions of siltartoxatug were prepared by diluting the stock solution in the vehicle to obtain the target drug concentrations. Concentration of the prepared dosing solutions was verified to be in the range of 98%–102% of the nominal concentrations by using a validated UV spectrophotometric method, which is fully complied with the predefined acceptance criteria.

### 2.2. Animals

All applicable portions of the study conformed to regulations and guidelines regarding animal care and welfare by AAALAC and China [20, 21].

Twenty‐four healthy naive cynomolgus monkeys, sourced from Guangdong Blooming‐Spring Biological Technology Co., Ltd., a qualified animal breeding facility, were enrolled in this study conducted in the animal facility of WuXi AppTec. Animals were each assigned with the unique identification numbers marked on the chest and recorded on the corresponding cage cards.

The animals were housed in controlled environmental chambers with relative humidity of 40%–70% and temperature at 18°C–29°C along with an air exchange rate of 10–20 cycles per hour. A 12‐hour light/dark cycle was implemented throughout the study, with exceptions permitted only for necessary experimental procedures.

All animals had ad libitum access to fresh purified drinking water and were fed a certified diet (120 g per feeding, twice daily). Fresh fruits were provided daily for nutritional enrichment.

### 2.3. Drug Administration

A total of twelve male and twelve female cynomolgus monkeys were randomized into four groups (Table 1), with three animals per sex in each group. Monkeys in Group 1 received a single IV dose of siltartoxatug at 1 mg/kg. Monkeys in Groups 2, 3, and 4 received a single IM dose of siltartoxatug at either 1, 3, or 10 mg/kg, respectively. Body weights, measured prior to dose administration and used to calculate the actual dose volume, ranged 2.53–3.12 kg in males and 2.39–3.09 kg in females.

**TABLE 1:** Study groups, route of administration, and sampling time.

Group	Animal number^∗^	Dosage	Route	Sampling time
1	C1001, C1002, C1003; C1501, C1502, C1503	1 mg/kg	IV infusion (30 min)	PK: Pre‐dose, 1, 2, 4, 8, 24, 48, 72, 96, 168, 240, 336, 504, 672, 1008, and 1344 h post‐dose. ADA: Pre‐dose, 168, 336, 504, 672, 1008, and 1344 h post‐dose.
2	C2001, C2002, C2003; C2501, C2502, C2503	1 mg/kg	IM	
3	C3001, C3002, C3003; C3501, C3502, C3503	3 mg/kg	IM	
4	C4001, C4002, C4003; C4501, C4502, C4503	10 mg/kg	IM	

^∗^Female: 001–003; male: 501–503.

### 2.4. Clinical Observation

Cage‐side observations for the general health status and physical appearance of the animals were conducted prior to dosing, post‐dosing, and at each sample collection time point. All abnormal clinical signs identified during the entire study period were recorded in detail.

### 2.5. Blood Sampling

Blood samples, not less than 0.8 mL for each, for PK and ADA analysis were collected into coagulant‐containing tubes at predefined time points, as detailed in Table 1. After incubation at room temperature for 30 min, sera were isolated by centrifugation of the coagulated blood samples at 4°C at 3000 × *g* for 10 min and aliquoted into duplicated vials: one for analysis and the other for backup. All samples were snap‐frozen in dry ice and stored at −60°C or below.

### 2.6. Bioanalysis and PK Data Analysis

Method validation and sample testing for both PK and ADA analysis were all performed in labs compliant with Good Laboratory Practice (GLP) [22–24].

An enzyme‐linked immunosorbent assay (ELISA) method was developed to specifically determine the concentration of siltartoxatug. Goat anti‐human IgG Fc (Sino Biological) was used to coat ELISA plates to capture siltartoxatug in the serum samples, and HRP‐labeled TBA002‐Target was used for detection. TBA002‐Target,a recombinant protein comprising the A and B fragments of tetanus toxin, retains the conformational epitope targeted by siltartoxatug. Method validation was performed following bioanalytical method validation guidelines issued by EMA, FDA, and NMPA [25–27]. Validation parameters included calibration range, accuracy and precision, dilution linearity and hook effect, selectivity (matrix effect and hemolysis), robustness, specificity, stability, and parallelism.

Serum concentrations of siltartoxatug were subjected to a noncompartmental PK analysis using Phoenix WinNonlin software (Version 6.3; Pharsight, Mountain View, CA). The linear/log trapezoidal rule was applied in calculating the PK parameters. Serum concentration values below the lower limit of quantification (BLQ) occurring prior to the first measurable concentration were assigned a value of zero. All other BLQ values were excluded from the analysis.

### 2.7. Immunogenicity Assay and ADA Testing

In compliance with FDA and EMA guidelines [28–30], a risk‐based, multitiered ADA testing approach was implemented to characterize ADA immune responses in this animal study.

An exclusive electrochemiluminescence (ECL) method on the Meso Scale Discovery (MSD) platform was developed for the study. Biotinylated‐siltartoxatug was immobilized on streptavidin‐precoated MSD plates. Serum ADAs bound to immobilized biotinylated‐siltartoxatug via one Fab arm and cross‐linked with ruthenylated‐siltartoxatug via the other arm, forming a sandwich immune bridge complex. The ECL signal emitted by excited ruthenium label was proportional to the serum ADA concentration. The assay positive control was a polyclonal anti‐idiotype antibody against siltartoxatug, which was prepared through repeated immunizations of New Zealand white rabbits with the F (ab)’ 2 of siltartoxatug by GenScript Biotech.

For method validation, serum samples from 25 treatment‐naïve cynomolgus monkeys, with and without siltartoxatug spiking, were tested repeatedly by different analysts over multiple days. The signal values obtained from unspiked serum samples were used to calculate the screening cut‐point factor (SCPF), which was established to achieve a false‐positive rate of approximately 5% to minimize the risk of false‐negative result. Meanwhile, the signal inhibition rate induced by siltartoxatug spiking was utilized to calculate the confirmatory cut‐point (CCP), with a predefined false‐positive rate of approximately 1%, to confirm the specificity of ADA signal for siltartoxatug. In addition, a comprehensive panel of validation parameters, including precision, sensitivity, drug tolerance, robustness, hook effect, selectivity (including matrix effect and hemolysis impact), and sample stability, was fully evaluated to verify the reliability of the established ADA assay method.

During sample testing, all unspiked study samples were initially screened with the SCPF‐derived threshold. Samples yielding positive screening results were further tested with siltartoxatug spiking, and the CCP was applied. Ultimately, all confirmed ADA‐positive samples underwent titration analysis to quantify the magnitude of the siltartoxatug‐induced immune response.

## 3. Result

### 3.1. Clinical Observations

All animals well tolerated siltartoxatug at the specified dosing levels throughout the entire study period. No adverse effects were observed in any of the tested animals during the in‐life phase of the study.

### 3.2. The Validation Results of PK Assay

A total of 17 analytical batches were conducted during the PK method validation, and all batches as well as validation samples met the acceptance criteria. The established calibration range was 10.0 to 1000 ng/mL. 10.0 (LLOQ, lower limit of quantification), 25.0 (LQC, low quality control), 110 (MQC, medium quality control), 750 (HQC, high quality control), and 1000 (ULOQ, upper limit of quantification) ng/mL were utilized during method validation. LQC, MQC, and HQC were utilized during sample testing. For accuracy and precision, intra‐assay results showed an accuracy (Bias%) range of −11.5%–15.2% and a precision (coefficient of variation, CV%) range of 0.6%–12.1%. Inter‐assay results demonstrated an accuracy (Bias%) range of −7.3%–8.0% and a precision (CV%) range of 3.6%–9.3%. The total error was determined to be 8.8%–15.5%. This method was confirmed to be specific for siltartoxatug detection in monkey serum, without matrix effects (including hemolysis interference) observed. It exhibited good robustness, and no hook effect was observed even at concentrations up to 300,000 ng/mL. The dilution factor was validated up to 15,000. Stability tests indicated that siltartoxatug remained stable in monkey serum after 7 freeze‐thaw cycles, or 24‐h storage at room temperature or 2°C–8°C, or 90‐day storage at ≤ ‐60°C. Finally, parallelism—reflecting the accuracy of diluted real samples—was demonstrated using incurred samples.

In summary, the PK assay employed in this study has been successfully validated for measuring the siltartoxatug serum concentration in cynomolgus monkey samples.

### 3.3. The Validation Results of ADA Assay

The floating SCPF and the CCP were determined to be 1.23 and 19%, respectively. The assay exhibited robust performance across multiple variable conditions, including different incubation periods, reagent lots, plate shakers, analysts, and assay days. The method sensitivity was determined to be 16.115 ng/mL for the positive control antibody in neat serum. No hook effect was observed even at a positive control antibody concentration up to 40,000 ng/mL. All serum lots tested, including hemolyzed serum samples, successfully passed the selectivity evaluation.

Detailed precision data were as follows. In the screening assay, intra‐assay precision (CV%) ranged 2.4%–13.7%; inter‐assay precision (CV%) ranged 6.7%–13.2%. In the confirmatory assay, the intra‐assay precision (CV%) ranged 0.4%–5.0%; inter‐assay precision (CV%) ranged 0.9%–5.7%.

In addition, drug tolerance tests demonstrated that the positive control antibody maintained detectable in the presence of siltartoxatug at the following threshold: 700 μg/mL siltartoxatug when the ADA concentration was 2000 ng/mL, 400 μg/mL siltartoxatug at 1000 ng/mL ADA, 350 μg/mL siltartoxatug at 500 ng/mL ADA, and 125 μg/mL siltartoxatug at 97 ng/mL ADA. Stability tests indicated that the positive control antibody remains stable in monkey serum after 7 freeze‐thaw cycles, or 24‐h storage at 2°C–8°C or room temperature, or 3‐month storage at ≤ ‐60°C.

In summary, the ADA assay employed in this study has been successfully validated for measuring the anti‐siltartoxatug antibody in cynomolgus monkey samples.

### 3.4. Siltartoxatug Pharmacokinetics in Monkey Serum After IV Infusion and IM Administration

PK profiles at all dose levels were plotted separately for male and female cynomolgus monkeys (Figure 1). For monkeys receiving IV administration, serum concentrations of siltartoxatug peaked immediately post‐dosing, followed by a brief marked decline, and subsequent slow decrease (Figure 1A). In contrast, for those receiving IM administration, serum concentrations increased rapidly, peaked at 2–96 h post‐dose, and then declined slowly after the T_max_ (time to maximum serum concentration; Figure 1B). No significant sex differences in the maximum serum concentration (C_max_) or the area under the serum concentration‐time curve from time zero to the last quantifiable concentration (AUC_0-last_) were observed across any dosing groups (Table 2). Therefore, PK profiles were further plotted using sex‐aggregated data (Figure 2), and corresponding PK parameters were calculated (Table 3).

**FIGURE 1:**
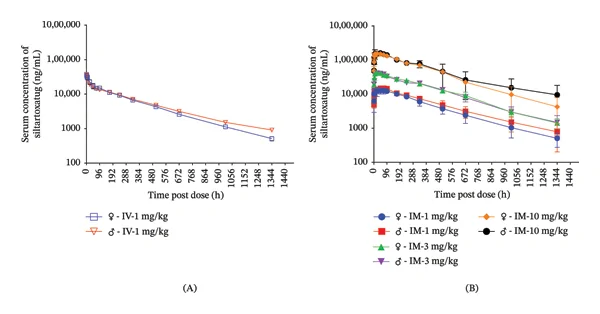
Mean serum concentration profiles in male or female cynomolgus monkeys after a single dose of siltartoxatug. (A) IV at the dose level of 1 mg/kg. (B) IM at the dose level of 1, 3, and 10 mg/kg, respectively. ♂: male; ♀: female. Data are shown as mean ± S.D.

**TABLE 2:** Analysis of sex differences in systemic exposure to siltartoxatug in cynomolgus monkeys.

Dose route	Dose level (mg/kg)	C_max_ ratio (female/male)	AUC_0-last_ ratio (female/male)
IV infusion	1.00	1.06	1.05
IM	1.00	1.16	1.20
IM	3.00	0.95	0.95
IM	10.0	1.05	1.09

*Note:* The ratio of C_max_ or AUC_0-last_ values ranging 0.50–2.00 indicates the absence of a significant sex difference.

**FIGURE 2:**
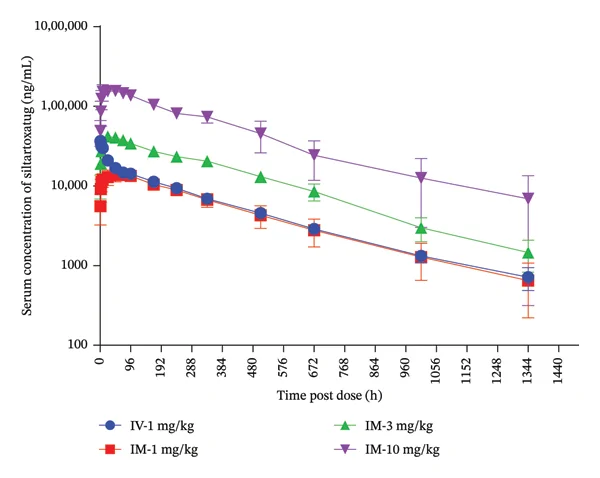
Mean serum concentration profiles in cynomolgus monkeys after a single dose of siltartoxatug. Data are calculated using sex‐combined serum concentrations and shown as mean ± S.D.

**TABLE 3:** Mean PK parameters in cynomolgus monkeys after siltartoxatug administration.

Dose (mg/kg), route	1, IV	1, IM	3, IM	10, IM
C_max_ (μg/mL)	36.8 ± 3.9	14.4 ± 2.3	42.5 ± 3.5	170.0 ± 21.3
C_1 h_ (μg/mL)	36.7 ± 4.1	5.6 ± 2.3	10.3 ± 3.4	49.0 ± 17.4
C_1344 h_ (μg/mL)	0.7 ± 0.2	0.6 ± 0.4	1.5 ± 0.6	6.9 ± 6.6
T_max_ (h)	1.17 ± 0.41	64.3 ± 35.4	33.3 ± 17.1	29.3 ± 15.7
t_1/2_ (h)	319 ± 60	298 ± 50	281 ± 79	288 ± 177
Vd (mL/kg)^a^	56.0 ± 7.2	69.2 ± 8.9	69.4 ± 13.8	58.7 ± 24.8
CL (mL/h/kg)^b^	0.1404 ± 0.00834	0.1638 ± 0.03108	0.1752 ± 0.02400	0.1608 ± 0.03984
AUC_0-last_ (μg·h/mL)	6810 ± 354	5990 ± 1010	16800 ± 1980	61200 ± 11600
AUC_0-inf_ (μg·h/mL)	7150 ± 414	6290 ± 1260	17400 ± 2300	65500 ± 16400
Bioavailability (%)^c^	—	88.0	—	—

*Note:* Data are shown as mean ± S.D. “—” means not calculated. AUC_0-inf_, area under the curve from time zero to infinity; CL, rate of clearance; CL/F, apparent clearance of drug after IM administration; T_max_, time to maximum serum concentration; t_1/2_, terminal half‐life; Vd, apparent volume of distribution; Vd_ss_, volume of distribution at steady state; Vz/F, apparent volume of distribution based on the terminal phase after IM administration.

^a^Specifically, it is Vd_ss_ for the IV group and Vz/F for the IM groups.

^b^Specifically, it is CL/F for the IM groups.

^c^Bioavailability was calculated using AUC_0-inf_ and nominal dose.

Absolute bioavailability of siltartoxatug at the 1 mg/kg IM dose was 88.0%, calculated relative to the dose‐matched 1 mg/kg IV reference group, demonstrating extensive absorption at this IM dose level. For the three IM doses (1, 3, and 10 mg/kg), dose‐normalized C_max_ and AUC_0-last_ ratios across doses were 1:0.99:1.18 and 1:0.93:1.02, respectively, demonstrating linear, dose‐proportional systemic exposure over the investigated IM dose range. Mean T_max_ values exhibited a shortening tendency with increasing IM dose, and substantial inter‐individual variability was observed consistently across all IM groups. Three key PK parameters were comparable across all tested IM doses and the IV reference group: mean volume of distribution (Vd_ss_ for IV group, Vz/F for IM groups) ranged from 56.0 to 69.4 mL/kg, mean systemic clearance (CL for IV group, CL/F for IM groups) ranged from 0.1404 to 0.1752 mL/h/kg, and mean terminal elimination half‐life (t_1/2_) ranged from 281 to 319 h (11.7–13.3 days), indicating that once siltartoxatug reaches systemic circulation, its systemic distribution and elimination profiles are comparable between IV and IM administration and remain consistent across the tested IM dose range.

### 3.5. Anti‐Siltartoxatug Antibodies in Cynomolgus Monkeys After IV Infusion and IM Administration

Of the 24 animals tested in this study, 3 exhibited positive ADA responses: one animal (C2503) in Group 2 (1/6) receiving 1 mg/kg siltartoxatug, and two animals (C4002 and C4501) in Group 4 (2/6) receiving 10 mg/kg siltartoxatug. ADA titers ranged 2–73.

## 4. Discussion

In the bioanalysis of mAb drugs, it is crucial to be aware of the specific state of analyte—either “total drugs” or “free drugs” [31]. For siltartoxatug, its therapeutic target is the exogenous tetanus toxin, which is not endogenously present in monkey serum. As a direct consequence, all siltartoxatug molecules in the serum exist in an unbound, free, and pharmacologically active state. Thus, the ELISA method in this study, which directly quantified “free siltartoxatug,” simultaneously provided concentration results of “total siltartoxatug.”

False negative results, which are unacceptable for immunogenicity assessment [28], may be induced by excessive drug concentrations. Therefore, drug tolerance—the assay’s capacity to detect ADA in the presence of expected high drug concentration—must be evaluated during method validation. Our ADA assay validation showed that 97 ng/mL could tolerate siltartoxatug concentrations up to 125 μg/mL, which is higher than 116 μg/mL, the highest serum siltartoxatug concentration from 168 h post‐dose (the first post‐dose ADA sampling time) onwards for all animals in this study, indicating that for all monkey samples, the positive control antibody at concentrations as low as 97 ng/mL would not produce false negative results. FDA recommends a minimum sensitivity of 100 ng/mL [30], confirming that our established ADA assay was competent for this animal study.

Although absolute bioavailability was not directly determined for the mid‐ and high‐dose IM groups due to the absence of corresponding IV data, the 88.0% bioavailability established at the low dose, combined with the linear and dose‐proportional systemic exposure observed across all IM dose levels, strongly suggests that absorption of siltartoxatug via this extravascular route remained comparably extensive at the higher doses in monkeys [32]. Detectable serum concentrations (5.6 ± 2.3 μg/mL) were achieved as early as 1 h after IM administration at 1 mg/kg, confirming early initiation of systemic absorption. While the standard deviations for most IM PK parameters were modest, T_max_ exhibited markedly larger inter‐animal variability, pointing to prominent variability in absorption timing. This is not unexpected for a mAb given intramuscularly, as absorption of large molecules occurs primarily via the lymphatic system [33], where drainage kinetics can vary considerably among individuals. By contrast, magnitude‐related endpoints such as overall systemic exposure were comparatively well‐controlled. Notably, T_max_ decreased with increasing dose, suggesting faster absorption at higher doses, although this did not compromise the overall dose‐proportional exposure profile. Thus, while inter‐individual variability in absorption timing exists, the overall absorption profile—characterized by high bioavailability and rapid onset—is favorable. With appropriate dose optimization informed by population‐based PK studies, this IM formulation has the potential to enable all subjects to attain protective concentrations shortly after administration.

The observed apparent volume of distribution (Vz/F) following IM administration of siltartoxatug aligns with what is expected for mAb preparations [17, 34, 35]. Moreover, this relatively low volume of distribution (< 0.07 L/kg) suggests that the drug remains largely confined to the vascular compartment in monkeys [36], which is advantageous for maintaining therapeutic concentrations in systemic circulation. However, this advantageous property awaits confirmation in subsequent clinical studies.

The FcRn binding is a key determinant of IgG pharmacokinetics. The dissociation constants for siltartoxatug binding to human FcRn were approximately 100 nM at pH 6.0 and 10 μM at pH 7.4 (unpublished data), values comparable to those previously reported for non‐Fc–engineered human mAbs [37]. Consistent with this binding profile, the clearance and t_1/2_ observed in this study fell within the known ranges for wild‐type human IgG in cynomolgus monkeys [17, 38]. Notably, most therapeutic mAbs undergo target‐mediated drug disposition (TMDD), a typical nonlinear clearance pathway that accelerates elimination and reduces half‐life [17]. However, siltartoxatug lacks endogenous pharmacological targets and TMDD is absent. As a result, in contrast to most therapeutic antibodies targeting endogenous antigens, siltartoxatug displays favorable and consistent low clearance and sustained half‐life across all tested dose levels. Collectively, the preserved native FcRn binding capacity and the lack of TMDD‐driven elimination underpin the robust dose‐proportional and linear PK profiles of siltartoxatug. A short‐term PK study in mice with IM injection of siltartoxatug at 0.05–5 mg/kg (unpublished data) also showed dose‐proportional systemic exposure. The linearity in pharmacokinetics observed over such wide dose ranges across species further supports reliable cross‐species PK extrapolation, which facilitates the safe and efficient selection of starting doses and design of dose escalation strategies in first‐in‐human trials [39, 40].

The typical incubation period for tetanus is approximately 8 days on average, ranging from 1 to 21 days [41]. Generally, longer incubation periods are observed when the injury site is located further from the central nervous system, while shorter incubation periods are associated with more severe disease and an increased risk of mortality. Therefore, both rapid attainment of effective serum concentrations/titers of anti‐toxin antibodies and sustained maintenance of such levels are critical for adequate tetanus prophylaxis. In this animal study, at 1 h after 1 mg/kg IM administration, the siltartoxatug serum concentration equates to 2.24 IU/mL based on the potency results of the toxin‐challenging study in mice [8], which is 224‐fold above the cross‐species protective level of 0.01 IU/mL [18, 42, 43]. Comparatively, a clinical study [44] showed that the mean serum titers of HTIG were 0.010, 0.017, 0.022, 0.032, and 0.014 IU/mL at 1, 2, 3, 7, and 14 days post IM administration of HTIG, respectively. Another clinical study [45] showed that only 83% of subjects had an HTIG titer > 0.01 IU/mL at 1‐day post‐administration of HTIG. Furthermore, even at 1344 h (57 days) post‐administration of 1 mg/kg, the serum concentrations of siltartoxatug remained at about 0.6 μg/mL, equivalent to 0.24 IU/mL, well above the protective threshold. Nevertheless, a single dose of HTIG typically contains ∼150–500 mg of proteins [9, 10], which is equivalent to ∼2.5–8 mg/kg of exogenous blood proteins for a 60‐kg individual. Together, IM‐administered siltartoxatug exhibited rapid achievement of effective levels with significantly lower exogenous protein burden, along with prolonged maintenance in monkeys, indicating that siltartoxatug can potentially provide quicker, longer‐lasting and safer protection in the clinic compared to HTIG.

As expected, siltartoxatug as a recombinant native human mAb has been demonstrated to have low and even below the detection limits of ADA responses in monkeys in this study. Although the induction of ADA in animals is not predictive of the potential for ADA formation in humans [15], the fully human origin of siltartoxatug supports the expectation of minimal immunogenicity in humans [46]. Thus, the ADA effect could be less concerned for the dosing regimen design in human trials. Clinical trials of siltartoxatug have indeed validated this premise, in which the ADA incidence was as low as 4.0% with titers ranging from 1.0 to 26.3 (unpublished data).

In conclusion, siltartoxatug exhibited a favorable PK profile in cynomolgus monkeys. Key characteristics included rapid attainment of protective drug levels, prolonged effective exposure, linear pharmacokinetics, and minimal ADA responses. These favorable pharmacological properties originate from its distinctive molecular design. These findings underpinned greater predictability for its clinical‐stage development and suggested promising clinical outcomes. Meanwhile, the nonclinical PK and immunogenicity data satisfied key global regulatory requirements for initiating first‐in‐human trials. On this basis, the investigational new drug (IND) application for siltartoxatug was approved by U.S. FDA and China NMPA in 2021, and marketing approval was granted by NMPA in 2023 following the positive Phase III trial results [47].

## Author Contributions

Xia Zhou, Zhenpeng Fu, Weijun Ye, Jason Li, and Xiaoyi Liu designed the study. Xia Zhou, Fei Zhou, Zhenpeng Fu, Weijun Ye, and Xiaoyi Liu analyzed the data. Xia Zhou, Fei Zhou, Guo Li, Jason Li, and Xiaoyi Liu prepared the manuscript. Weihong Zheng, Hua‐Xin Liao, and Shaojie Wang contributed to data interpretation and critical review of the paper.

## Funding

This work was supported by Zhuhai Innovative and Entrepreneurial Research Team Program, China (no. ZH01110405180040PWC).

## Disclosure

All authors approved the submission of this paper for publication.

## Conflicts of Interest

Xia Zhou, Fei Zhou, Zhenpeng Fu, Weijun Ye, Guo Li, Jason Li, Weihong Zheng, Hua‐Xin Liao, and Xiaoyi Liu are employees of Trinomab at the time of the study. The other authors declare no conflicts of interest.

## Data Availability

The data that support the findings of this study are available from the corresponding authors upon reasonable request.
